# Prognostic value of albumin-bilirubin score in pancreatic cancer patients after pancreatoduodenectomy with liver metastasis following radiofrequency ablation

**DOI:** 10.3389/pore.2023.1611175

**Published:** 2023-05-30

**Authors:** Lei Zhang, Xuefei Zhang, Bolin Wu, Xue Han, Cunli Guo, Bo Li, Hui Jing, Wen Cheng

**Affiliations:** ^1^ Department of Ultrasound, Harbin Medical University Cancer Hospital, Harbin, China; ^2^ Interventional Ultrasound Ward, Harbin Medical University Cancer Hospital, Harbin, China

**Keywords:** chemotherapy, pancreatic cancer, liver metastasis, radiofrequency ablation, pancreatoduodenectomy

## Abstract

**Objective:** The current study aimed to investigate the prognostic value of albumin-bilirubin (ALBI) score in predicting clinical outcomes of pancreatic cancer patients after pancreatoduodenectomy with liver metastasis following radiofrequency ablation.

**Methods:** This retrospective study included 90 pancreatic cancer patients after pancreatoduodenectomy with liver metastasis from January 2012 to December 2018. In this study, the Chi-square or Fisher’s exact tests, the receiver operating characteristic (ROC) curve, Kaplan-Meier method and Log-rank test, univariate and multivariate Cox proportional hazard regression analyses, nomogram, calibration curves and decision curve analysis were used for all statistical analysis.

**Results:** We analyzed the optimal cut-off value of ALBI by ROC curve, and the optimal cut-off value was −2.60. According to ALBI score, these patients were divided into two groups: low ALBI group (*n* = 33) and high ALBI group (*n* = 57). Patients with low ALBI score was significantly related to longer progression free survival (PFS) (*p* = 0.0002, HR: 3.039, 95% CI: 1.772–5.210) and overall survival (OS) (*p* = 0.0005, HR: 2.697, 95% CI: 1.539–4.720). The 1-, 3-, and 5-year PFS and OS rates in low ALBI group were higher than those in high ALBI group. ALBI was a potential independent prognostic factor for pancreatic cancer patients after pancreatoduodenectomy with liver metastasis following radiofrequency ablation. Moreover, the nomogram was used to predict the 1-, 3-, and 5-year survival probabilities of PFS and OS. The calibration curve shown that the prediction line matched the reference line well for postoperative 3-year PFS and OS. The DCA shown that nomogram model was better than the only ALBI, and indicated the ability for clinical decision-making, especially in 1-year PFS, and 3-, 5-year OS.

**Conclusion:** ALBI is a potential independent factor for PFS and OS, and can predict the prognosis of pancreatic cancer patients after pancreatoduodenectomy with liver metastasis following radiofrequency ablation.

## Introduction

Pancreatic cancer is the fourteenth leading cause of cancer-related incidence and the seventh leading cause of cancer-related deaths all over the world [1]. As a result of the changes in lifestyle, the incidence has been increasing in developed countries, especially in western countries [2]. Surgery is the primary treatment for early and resectable pancreatic cancer. With the progress of surgical technology, adjuvant treatment, and the improvement of perioperative management are now continuously increasing [3]. However, due to delay diagnosis, ineffective treatment, no obvious symptom, the majority of the patients are not appropriate candidates for operation [4]. Despite the progress in the detection and treatment of pancreatic cancer, the 1-year survival rate is about 24%, and the 5-year survival rate is about 6% [5]. Liver is the most common site of metastasis, and synchronous liver metastasis reportedly occurs in approximately 50% of pancreatic cancer patients at the time of initial diagnosis, and postoperative liver metastasis accounts for 40%–90% [6, 7]. Although the simultaneous resection of pancreatic cancer and liver metastasis are technically feasible, and liver metastasis is still considered to be the main cause of death in patients with pancreatic cancer [8].

Radiofrequency ablation (RFA) is a non-surgical treatment for liver metastasis of pancreatic cancer with unresectable liver metastases or complications [9]. Compared with systemic chemotherapy, hepatectomy can increase the local disease control rate, improve progression free survival (PFS) and have better overall survival (OS) [10]. However, not all pancreatic cancer patients with liver metastases can benefit from hepatectomy due to the aggressive tumor behavior, limited surgical operation indications or frequent liver failure after liver resection [11]. In order to improve the survival outcomes of pancreatic cancer with liver metastases, the hepatic arterial infusion chemotherapy, systemic chemotherapy, RFA and radiotherapy have been practiced in the clinical setting [12–15].

Tumor associated systemic inflammation plays an important role in the development and metastasis of tumor cells [16]. Previous studies have revealed that systemic inflammation biomarkers, such as neutrophil-to-lymphocyte ratio (NLR), lymphocyte-to-monocyte ratio (LMR), and platelet-to-lymphocyte ratio (PLR), were closely associated with tumor prognosis [17, 18]. A novel inflammation-related marker, albumin-bilirubin (ALBI) score, calculated from albumin and bilirubin, has been identified for the first time as estimating the degree of liver function in patients with hepatocellular carcinoma [19]. ALBI has been gradually emerged as an independent predictor in some tumors, such as colon cancer, high-grade gliomas, and hepatocellular carcinoma [20–22]. However, there are few studies on the role of ALBI in pancreatic cancer after pancreatoduodenectomy with liver metastasis. Hence, the aim of our study was to investigate the association of ALBI with clinicopathological indicators and survival outcomes in pancreatic cancer patients after pancreatoduodenectomy with liver metastasis following radiofrequency ablation.

## Materials and methods

### Patients section

The inclusion criteria were as follows: 1) pancreatic cancer confirmed by histopathology after pancreatoduodenectomy; 2) positron emission tomography computer tomography (PETCT)/multi-detector computed tomography (MDCT) imaging diagnosis confirmed liver metastasis, and without extrahepatic diffusion; 3) single or multiple liver metastases (less than five lesions), and the maximum size of the largest liver lesion less than 5 cm; 4) Eastern Cooperative Oncology Group (ECOG) < 2 scores and Karnofsky (KPS) ≥70 scores, and could bear the risk of the treatment; 5) with complete follow-up data. The exclusion criteria were as follows: 1) with organ metastasis or other tumors; 2) with serious complications, such as infection, active bleeding, coagulation abnormalities; 3) with any severe comorbidities, such as respiratory failure, renal failure or heart failure; 4) with poor clinical compliance.

### Process of RFA and chemotherapy regimens

RFA therapeutic instrument with multiple probe approaches for the RFA operation. RFA was conducted under the guidance of B-type ultrasound. Intraoperative ultrasound and magnetic resonance imaging (MRI) were used to detect whether the liver metastases were completely necrotic by the latest guidelines of National Comprehensive Cancer Network (NCCN) and Chinese Society of Clinical Oncology (CSCO). According to the size and location of the tumor, single needle or multi needle electrodes with 2 or 3 cm tip were used during the operation. Depending on the size of the tumor, each ablation cycle lasted 6–12 minutes. Patients need to undergo one or more RFA treatments. At the end of the treatment, the puncture pathway was solidified to avoid bleeding during needle extraction. All hepatic metastasis were imaged by B-type ultrasound to detect whether abnormal enhancement or not. When all related adverse events of RFA were resolved and the liver metastases were confirmed to be completely removed, the patients were given systemic chemotherapy after RFA intervention for 2–4 weeks. The chemotherapy regimen included GO regimen (Gemcitabine and Oxaliplatin combination); GT regimen (Gemcitabine and Tegafur combination). All patients could tolerate the side effects of chemotherapy.

### Follow up

All patients were regularly followed up after RFA. The progression free survival (PFS) was defined as the time from RFA to intrahepatic or extrahepatic recurrence or progression. The overall survival (OS) was defined as the time from RFA to death or last follow up.

### Calculation of the ALBI score

The blood routine and biochemical tests were obtained at the first day of admission in our hospital. The albumin-bilirubin (ALBI) score was evaluated by albumin and direct bilirubin measurements, the direct bilirubin is in μmol/L, and albumin is in g/L. The ALBI score was as follows: ALBI score = 0.660× log10 direct bilirubin - 0.085×albumin. The ALBI score was referred to the previous literature [19]. According to the receiver operating characteristic (ROC) curve, we analyzed the optimal cut-off value of ALBI. In our study, we divided these patients into two groups: low ALBI group (≤−2.60) and high ALBI group (>−2.60).

### Statistical analysis

Baseline characteristics were described as numbers (%) for categorical variables. The Chi-square or Fisher’s exact tests were used to compare categorical variables. The Kaplan-Meier and Log-rank test were constructed to determine the survival curve. The hazard ratio (HR) and 95% Confidence Interval (CI) were performed to evaluate the association between ALBI score and prognosis. The univariate and multivariate Cox proportional hazard regression analyses were used to evaluate the potential independent factors. The prognostic nomogram were conducted according to the multivariate analyses. The calibration curves and decision curve analysis were performed to evaluate the predictive performance. All statistical analysis were performed by R (version 3.6.0), SPSS Statistics software (version 22.0) and GraphPad prism software (version 8.0), and a two-tailed *p* < 0.05 was considered statistically significant.

## Results

### Baseline clinicopathologic characteristics

Ninety pancreatic cancer after pancreatoduodenectomy with liver metastasis were recruited for this study. These patients were received RFA treatment, and 69 patients were received chemotherapy after RFA. There were 45 males and 45 females in this study. The mean age was 55 years, with the range from 34 to 73 years, and the median age was 57 years. Of all patients, 21 patients with poorly differentiated pancreatic cancer, 60 patients with moderately differentiated pancreatic cancer, and 9 patients with well differentiated pancreatic cancer, respectively. Adenocarcinoma is the main pathological type, accounting for 86.7% (78/90). The baseline clinicopathologic characteristics were listed in Table 1. According to ALBI score, these patients were divided into two groups: low ALBI group (*n* = 33) and high ALBI group (*n* = 57). Compared to these characteristics, ALBI was associated with underlying disease (*p* = 0.045) and liver metastases (*p* = 0.008).

**TABLE 1:** Characteristics of patients with liver metastasis after pancreatoduodenectomy.

Parameters	Level	Overall	Low ALBI	High ALBI	*p*
Cases (n)		90	33	57	
Sex	Male	45 (50.0)	15 (45.5)	30 (52.6)	0.662
	Female	45 (50.0)	18 (54.5)	27 (47.4)	
Age	<57	44 (48.9)	13 (39.4)	31 (54.4)	0.249
	≥57	46 (51.1)	20 (60.6)	26 (45.6)	
Differentiation	Poorly	21 (23.3)	7 (21.2)	14 (24.6)	0.460
	Moderately	60 (66.7)	21 (63.6)	39 (68.4)	
	Well	9 (10.0)	5 (15.2)	4 (7.0)	
Pathological type	Adenocarcinoma	78 (86.7)	30 (90.9)	48 (84.2)	0.563
	Mucinous carcinoma	12 (13.3)	3 (9.1)	9 (15.8)	
Underlying disease	Yes	28 (31.1)	15 (45.5)	13 (22.8)	0.045
	No	62 (68.9)	18 (54.5)	44 (77.2)	
Liver metastases	Single	32 (35.6)	18 (54.5)	14 (24.6)	0.008
	Multiple	58 (64.4)	15 (45.5)	43 (75.4)	
Size of liver metastases	≤2 cm	27 (30.0)	13 (39.4)	14 (24.6)	0.215
	>2 cm	63 (70.0)	20 (60.6)	43 (75.4)	
Chemotherapy	No	21 (23.3)	8 (24.2)	13 (22.8)	1.000
	Yes	69 (76.7)	25 (75.8)	44 (77.2)	

### Associations between blood parameters and ALBI score

The blood parameters were obtained before RFA. We analyzed blood parameters by median value. Compared with two groups, there were significant differences in carcinoembryonic antigen (CEA) (*p* = 0.033), aspartate aminotransferase (AST) (*p* = 0.002), total protein (*p* = 0.029), albumin (*p* = 0.012), total bilirubin (*p* = 0.029), direct bilirubin (*p* = 0.014), creatinine (*p* = 0.019), neutrophils (*p* = 0.001), respectively. The detail information were shown in Table 2.

**TABLE 2:** Associations between blood parameters and ALBI score.

Parameters	Level	Overall	Low ALBI	High ALBI	*p*
Cases (n)		90	33	57	
CA199	≤19.91	45 (50.0)	16 (48.5)	29 (50.9)	1.000
	>19.91	45 (50.0)	17 (51.5)	28 (49.1)	
CEA	≤0.45	50 (55.6)	13 (39.4)	37 (64.9)	0.033
	>0.45	40 (44.4)	20 (60.6)	20 (35.1)	
ALT	≤65.50	47 (52.2)	21 (63.6)	26 (45.6)	0.153
	>65.50	43 (47.8)	12 (36.4)	31 (54.4)	
AST	≤53.50	45 (50.0)	24 (72.7)	21 (36.8)	0.002
	>53.50	45 (50.0)	9 (27.3)	36 (63.2)	
r-GT	≤32.70	46 (51.1)	18 (54.5)	28 (49.1)	0.782
	>32.70	44 (48.9)	15 (45.5)	29 (50.9)	
Alkaline phosphatase	≤100.0	46 (51.1)	17 (51.5)	29 (50.9)	1.000
	>100.0	44 (48.9)	16 (48.5)	28 (49.1)	
Total protein	≤66.25	45 (50.0)	11 (33.3)	34 (59.6)	0.029
	>66.25	45 (50.0)	22 (66.7)	23 (40.4)	
Albumin	≤37.40	47 (52.2)	11 (33.3)	36 (63.2)	0.012
	>37.40	43 (47.8)	22 (66.7)	21 (36.8)	
Total bilirubin	≤11.94	45 (50.0)	22 (66.7)	23 (40.4)	0.029
	>11.94	45 (50.0)	11 (33.3)	34 (59.6)	
Direct bilirubin	≤5.79	46 (51.1)	23 (69.7)	23 (40.4)	0.014
	>5.79	44 (48.9)	10 (30.3)	34 (59.6)	
Creatinine	≤62.00	46 (51.1)	11 (33.3)	35 (61.4)	0.019
	>62.00	44 (48.9)	22 (66.7)	22 (38.6)	
Glucose	≤5.80	49 (54.4)	17 (51.5)	32 (56.1)	0.838
	>5.80	41 (45.6)	16 (48.5)	25 (43.9)	
Hemoglobin	≤112	46 (51.1)	15 (45.5)	31 (54.4)	0.550
	>112	44 (48.9)	18 (54.5)	26 (45.6)	
Platelet	≤175	46 (51.1)	16 (48.5)	30 (52.6)	0.873
	>175	44 (48.9)	17 (51.5)	27 (47.4)	
Lymphocyte	≤1.30	47 (52.2)	14 (42.4)	33 (57.9)	0.231
	>1.30	43 (47.8)	19 (57.6)	24 (42.1)	
White blood cell	≤8.22	45 (50.0)	21 (63.6)	24 (42.1)	0.080
	>8.22	45 (50.0)	12 (36.4)	33 (57.9)	
Neutrophils	≤8.22	47 (52.2)	25 (75.8)	22 (38.6)	0.001
	>8.22	43 (47.8)	8 (24.2)	35 (61.4)	
Red blood cell	≤3.85	57 (63.3)	24 (72.7)	33 (57.9)	0.238
	>3.85	33 (36.7)	9 (27.3)	24 (42.1)	
Prothrombin time	≤11.90	70 (77.8)	24 (72.7)	46 (80.7)	0.539
	>11.90	20 (22.2)	9 (27.3)	11 (19.3)	
Prothrombin activity	≤98.00	68 (75.6)	22 (66.7)	46 (80.7)	0.216
	>98.00	22 (24.4)	11 (33.3)	11 (19.3)	
INR	≤0.89	70 (77.8)	24 (72.7)	46 (80.7)	0.539
	>0.89	20 (22.2)	9 (27.3)	11 (19.3)	

CA199, carbohydrate antigen199; CEA, carcinoembryonic antigen; ALT, alanine aminotransferase; AST, aspartate aminotransferase; r-GT, r-glutamyltransferase.

### Univariate and multivariate analysis

**FIGURE 1:**
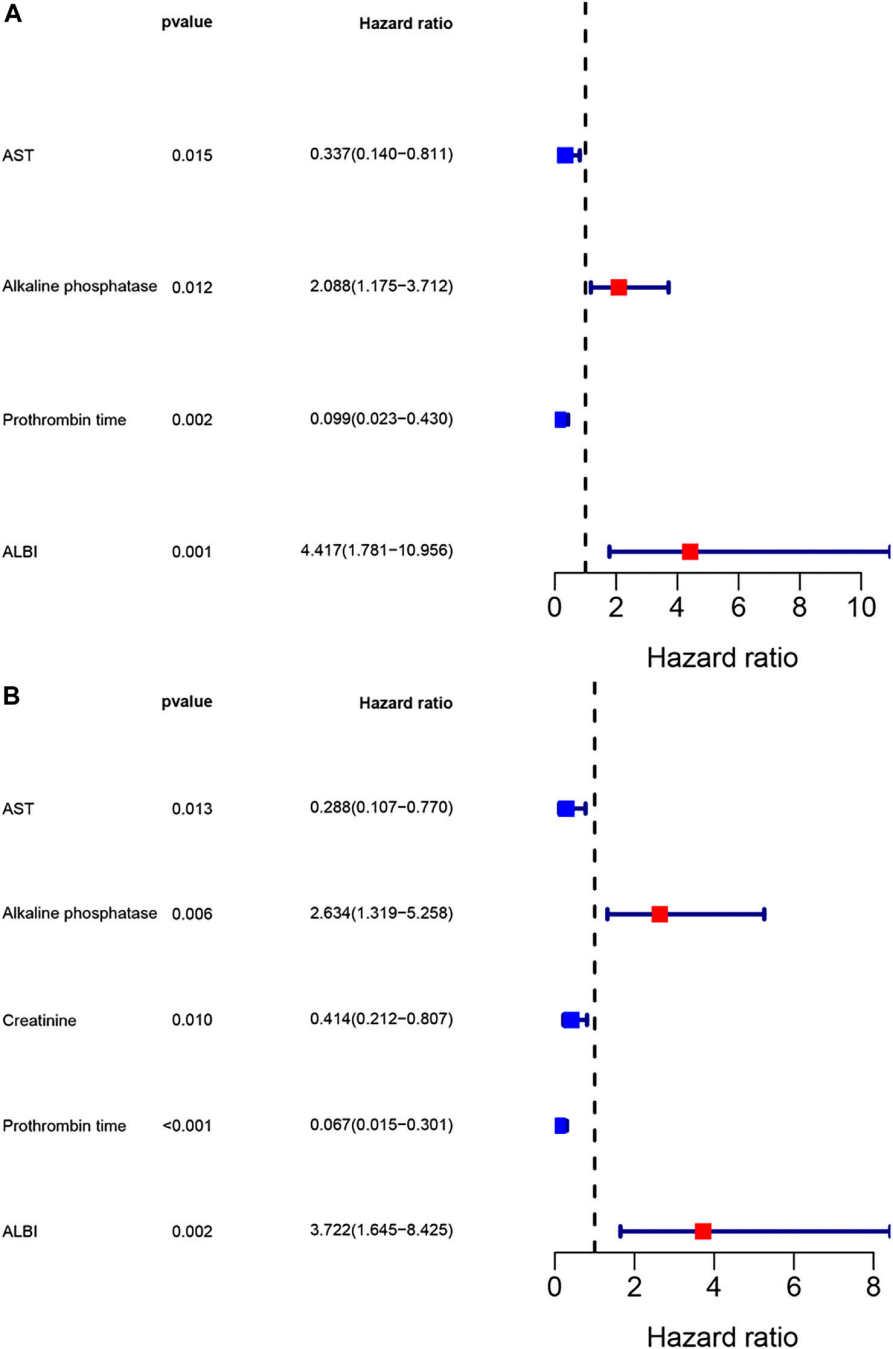
Forest plots for multivariate analysis. **(A)** Multivariate analysis for progression free survival; **(B)** Multivariate analysis for overall survival.

The univariate analysis revealed that sex, AST, alkaline phosphatase, creatinine, neutrophils, prothrombin time, ALBI and chemotherapy were related to the prognosis for PFS, however, the multivariate analysis revealed that AST, alkaline phosphatase, prothrombin time and ALBI were the potential independent prognostic factors for PFS. Moreover, the univariate analysis revealed that sex, pathological type, AST, alkaline phosphatase, creatinine, neutrophils, prothrombin time, ALBI and chemotherapy were related to the prognosis for OS, however, the multivariate analysis revealed that AST, alkaline phosphatase, creatinine, prothrombin time and ALBI were the potential independent prognostic factors for OS. The detail information was shown in Table 3. The multivariate analysis results were displayed using forest plots (Figure 1).

**TABLE 3:** Univariate and multivariate analysis of progression free survival and overall survival.

Characteristics	PFS						OS					
	HR	Univariate 95% CI	*p*	HR	Multivariate 95% CI	*p*	HR	Univariate 95% CI	*p*	HR	Multivariate 95% CI	*p*
Sex (Male vs. Female)	0.476	0.274–0.827	0.008	0.596	0.330–1.076	0.086	0.536	0.309–0.931	0.027	0.811	0.442–1.488	0.498
Age (<57 vs. ≥57 years)	1.127	0.656–1.937	0.665				1.108	0.645–1.906	0.710			
Differentiation (Poorly vs. Moderately + Well)	0.784	0.485–1.267	0.320				0.767	0.466–1.263	0.298			
Pathological type (Adenocarcinoma vs. Mucinous)	1.481	0.742–2.957	0.265				2.352	1.173–4.717	0.016	1.961	0.790–4.865	0.147
Underlying disease (Yes vs. No)	1.267	0.685–2.342	0.450				1.340	0.725–2.477	0.351			
Liver metastases (Single vs. Multiple)	1.098	0.621–1.943	0.747				1.249	0.706–2.211	0.444			
Size of liver metastases (≤2 vs. >2 cm)	1.067	0.592–1.923	0.829				1.043	0.579–1.880	0.888			
CA199 (≤19.91 vs. >19.91)	0.832	0.483–1.435	0.509				0.775	0.449–1.335	0.358			
CEA (≤0.45 vs. >0.45)	0.615	0.350–1.079	0.090				0.623	0.355–1.093	0.099			
ALT (≤65.50 vs. >65.50)	1.292	0.752–2.218	0.353				1.234	0.717–2.125	0.448			
AST (≤53.50 vs. >53.50)	1.868	1.068–3.270	0.029	0.337	0.140–0.811	0.015	1.914	1.092–3.353	0.023	0.288	0.107–0.770	0.013
r.GT (≤32.70 vs. >32.70)	1.157	0.674–1.986	0.597				1.090	0.636–1.870	0.754			
Alkaline phosphatase (≤100.0 vs. >100.0)	1.948	1.125–3.373	0.017	2.088	1.175–3.712	0.012	2.137	1.231–3.709	0.007	2.634	1.319–5.258	0.006
Total protein (≤66.25 vs. >66.25)	1.047	0.611–1.794	0.868				0.887	0.518–1.521	0.664			
Albumin (≤37.40 vs. >37.40)	1.155	0.673–1.982	0.602				1.327	0.773–2.276	0.305			
Total bilirubin (≤11.94 vs. >11.94)	1.430	0.830–2.461	0.197				1.421	0.826–2.446	0.204			
Direct bilirubin (≤5.79 vs. >5.79)	1.318	0.767–2.266	0.318				1.257	0.732–2.158	0.407			
Creatinine (≤62.00 vs. >62.00)	0.563	0.322–0.983	0.043	0.581	0.303–1.113	0.102	0.518	0.297–0.906	0.021	0.414	0.212–0.807	0.010
Glucose (≤5.80 vs. >5.80)	0.987	0.570–1.708	0.962				0.869	0.502–1.504	0.616			
Hemoglobin (≤112 vs. >112)	0.721	0.418–1.244	0.240				0.710	0.412–1.224	0.217			
Platelet (≤175 vs. >175)	1.113	0.649–1.909	0.698				0.983	0.572–1.687	0.949			
Lymphocyte (≤1.30 vs. >1.30)	0.984	0.574–1.687	0.953				1.127	0.657–1.933	0.664			
White blood cell (≤8.22 vs. >8.22)	0.866	0.504–1.490	0.604				0.808	0.470–1.389	0.441			
Neutrophils (≤6.21 vs. >6.21)	1.816	1.051–3.139	0.033	1.481	0.743–2.950	0.264	1.764	1.022–3.044	0.041	1.855	0.763–4.510	0.173
Red blood cell (≤3.85 vs. >3.85)	1.151	0.663–1.998	0.618				1.178	0.679–2.045	0.561			
Prothrombin time (≤11.90 vs. >11.90)	0.317	0.135–0.744	0.008	0.099	0.023–0.430	0.002	0.326	0.139–0.767	0.010	0.067	0.015–0.301	0.000
Prothrombin activity (≤98.00 vs. >98.00)	1.678	0.919–3.064	0.092				1.538	0.845–2.800	0.159			
ALBI (Low vs. High)	3.257	1.684–6.301	0.000	4.417	1.781–10.956	0.001	3.070	1.582–5.960	0.001	3.722	1.645–8.425	0.002
Chemotherapy (No vs. Yes)	0.421	0.189–0.935	0.034	1.621	0.411–6.386	0.490	0.425	0.191–0.947	0.036	2.027	0.496–8.291	0.325

### Survival analysis by ABLI score

**FIGURE 2:**
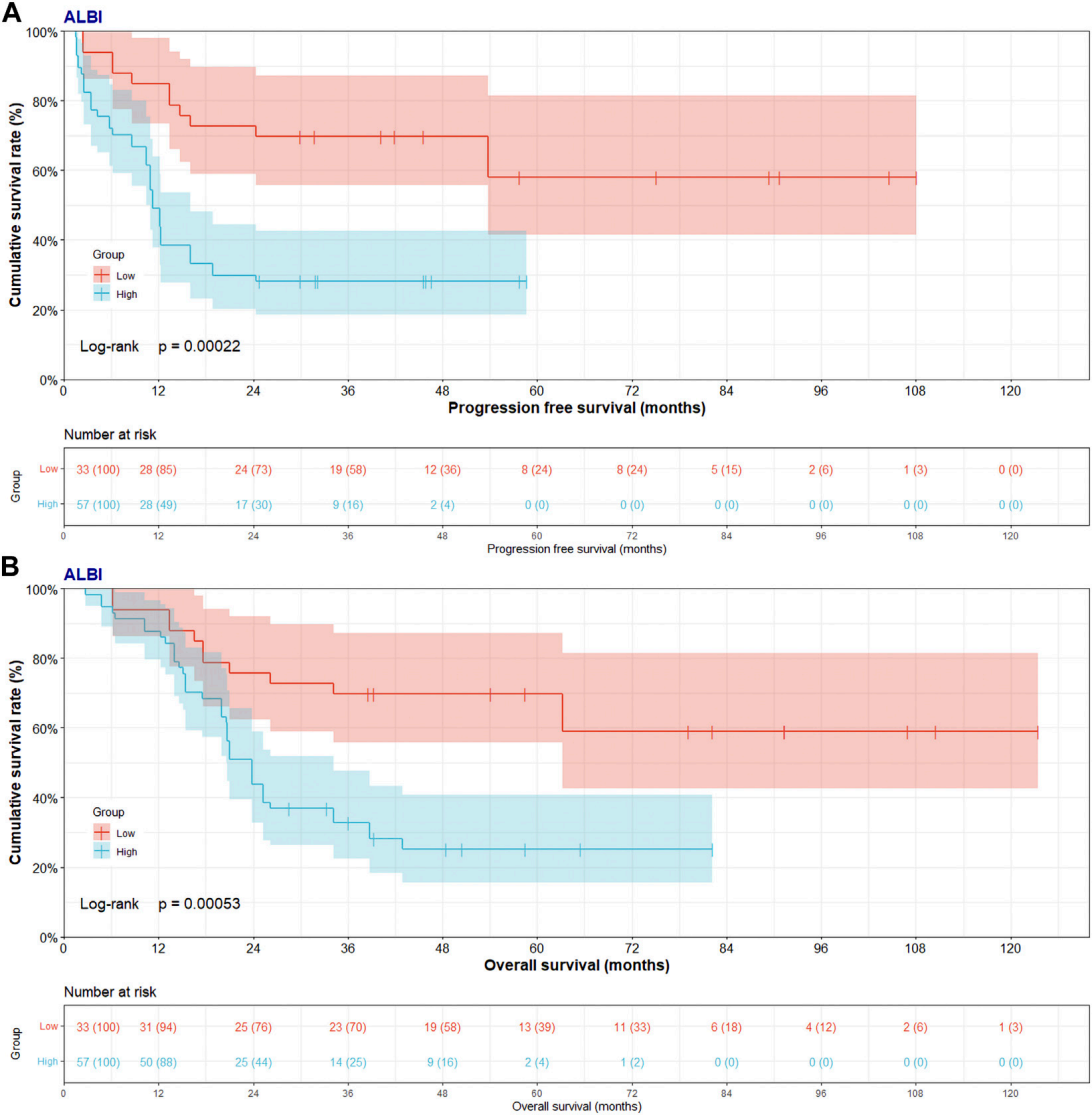
Progress free survival (PFS) and overall survival (OS) of patients with pancreatic cancer after pancreatoduodenectomy with liver metastasis treated with RFA by ALBI score. **(A)** Kaplan-Meier analysis of PFS for patients treated with RFA by ALBI score; **(B)** Kaplan-Meier analysis of OS for patients treated with RFA by ALBI score.

In low ABLI group, the median PFS and OS were 40.17 (95% CI: 29.97–53.80) and 54.07 (95% CI: 38.57–79.10) months. In high ABLI group, the median PFS and OS were 11.30 (95% CI: 10.47–16.00) and 23.83 (95% CI: 20.03–28.53) months. Compared with high ABLI group, the median PFS and OS in low ABLI group were survived longer and had better prognosis (PFS, *p* = 0.0002; OS, *p* = 0.0005) (Figure 2). Moreover, the 1-, 3- and 5-year PFS rates were 84.8% (95% CI: 0.735–0.980), 69.7% (95% CI: 0.557–0.873), 58.1% (95% CI: 0.414–0.815) in low ABLI group, and the 1-, 3- and 5-year PFS rates were 49.1% (95% CI: 0.377–0.640), 28.1% (95% CI: 0.185–0.425), 0% (95% CI: 0.000–0.000) in high ABLI group. Furthermore, the 1-, 3- and 5-year OS rates were 93.9% (95% CI: 0.861–1.000), 69.7% (95% CI: 0.557–0.873), 69.7% (95% CI: 0.557–0.873) in low ABLI group, and the 1-, 3- and 5-year PFS rates were 87.7% (95% CI: 0.796–0.967), 32.7% (95% CI: 0.225–0.477), 25.3% (95% CI: 0.156–0.408) in high ABLI group.

### Nomogram for PFS and OS

**FIGURE 3:**
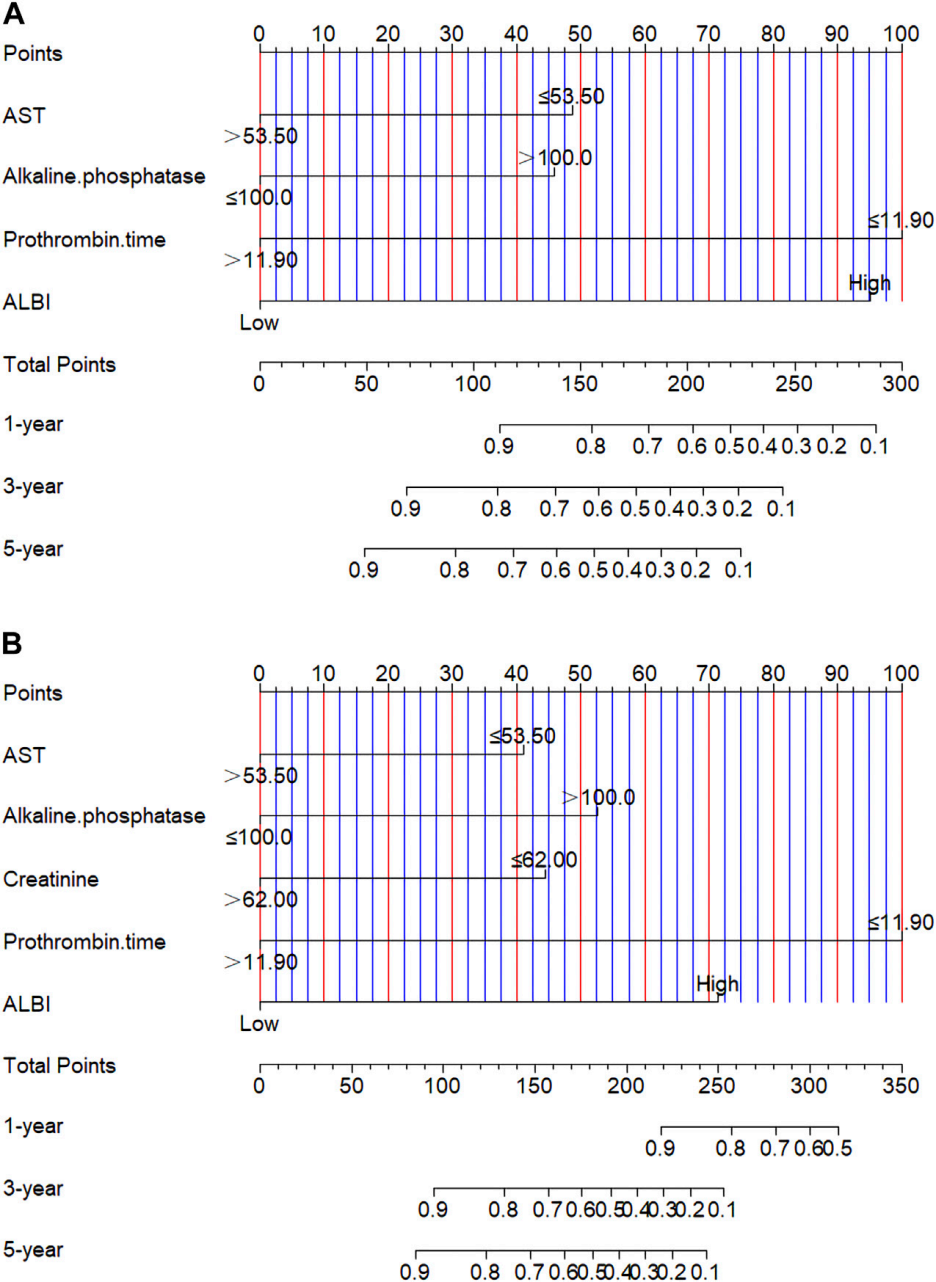
Nomogram for predicting progress free survival (PFS) and overall survival (OS). **(A)** Nomogram for predicting PFS; **(B)** Nomogram for predicting OS.

**FIGURE 4:**
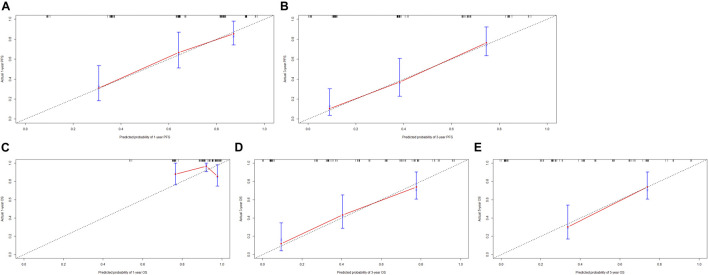
The calibration curves for predicting the 1-, 3-, 5-year PFS and OS rates. **(A)** The calibration curves for predicting the 1-year PFS rate; **(B)** The calibration curves for predicting the 3-year PFS rate; **(C)** The calibration curves for predicting the 1-year OS rate; **(D)** The calibration curves for predicting the 3-year OS rate; **(E)** The calibration curves for predicting the 5-year OS rate.

**FIGURE 5:**
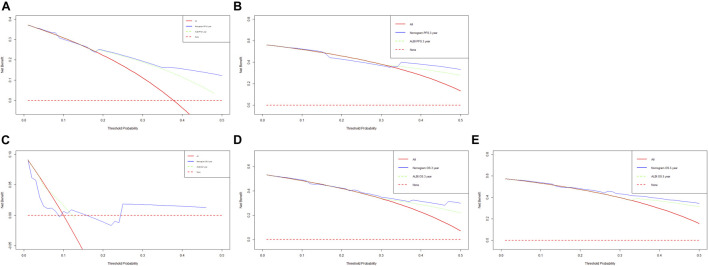
Decision curve analysis (DCA) of the nomogram (the independent prognostic factors by multivariate analysis) and only ALBI score for predicting progress free survival (PFS) and overall survival (OS). **(A)** DCA of the nomogram and treatment for predicting the 1-year PFS; **(B)** DCA of the nomogram and treatment for predicting the 3-year PFS; **(C)** DCA of the nomogram and treatment for predicting the 1-year OS; **(D)** DCA of the nomogram and treatment for predicting the 3-year OS; **(E)** DCA of the nomogram and treatment for predicting the 5-year OS.

Though the multivariate analysis, we constructed a nomogram for individualized assessment of PFS and OS. By the nomogram, every enrolled variable was imputed a weighted point, and the sum of the points could predict 1-, 3- and 5-year survival probabilities for PFS and OS. The nomogram for PFS had integrated AST, alkaline phosphatase, prothrombin time and ALBI; and the OS had integrated AST, alkaline phosphatase, creatinine, prothrombin time and ALBI (Figure 3). The patients with high grade were related to a lower survival probability. Moreover, we used the calibration curve to determine the nomogram for the predicted and actual probability of PFS and OS. The prediction line matched the reference line well for 3-year PFS and OS (Figure 4). Furthermore, we also used the decision curve analysis to determine the clinical utility between nomogram (the potential independent prognostic factors by multivariate analysis) and ALBI by quantifying the net benefits at different threshold probabilities. The nomogram model was better than the only ALBI, and shown the ability for clinical decision-making, especially in 1-year PFS, and 3-, 5-year OS (Figure 5).

## Discussion

Metastatic disease is the cause of over 90% of solid tumor related mortality, however, it is still the least known component of cancer pathogenesis [23, 24]. Liver is highly metastatic organ, as for its unique and diverse structure, cell composition enables the liver to undertake many special functions, and liver metastases are more common than primary hepatic tumors [25, 26]. The biological characteristics of liver, especially its hemodynamic characteristics and unique microenvironment, make the liver essentially suitable for disseminated tumor cells. The primary tumors, such as breast cancer, colorectal cancer, neuroendocrine tumors, prostate carcinomas, gastric cancer, uveal melanoma and pancreatic cancer, were easy to spread to the liver [27–33]. Moreover, the studies indicated that the prognosis of patients with liver metastasis was worse than that of patients without liver metastasis [8]. At present, about 30%–70% of patients was to die of liver metastasis disease, and increased the costs and spending obviously [34]. Various treatments, including surgery (liver resection), chemotherapy, RFA, trans arterial chemoembolization (TACE), hepatic arterial infusion chemotherapy, have been used in clinical practice. Pancreatic cancer has a very poor prognosis. Pancreatoduodenectomy is the main treatment approach, however, less than 20% of patients can survive more than 5 years [35]. Many pancreatic cancer patients have liver metastasis even after pancreatoduodenectomy. The chemotherapy are used for the treatment of pancreatic cancer or after pancreatoduodenectomy [36, 37]. However, as a result of the serious adverse reactions, damage the quality of life, not all patients can benefit from chemotherapy [38, 39]. Some studies have pointed out that RFA was an appropriate choice for patients with solitary liver metastasis of malignant tumors, and could effectively inhibit the growth and metastasis of cancer cells [40, 41]. However, the effectively predictors which used to evaluate the prognosis of pancreatic cancer was still not clear. Therefore, it is necessary to find prognostic biomarkers for pancreatic cancer patients after pancreatoduodenectomy with liver metastasis.

ALBI is a new marker of malignant tumor recently described, which is specifically characterized by a comprehensive assessment of nutritional status and liver function. Nevertheless, few studies have studied the relationship between ALBI and prognosis in pancreatic cancer. It is reported that low ALBI is a positive indicator for better survival in pancreatic cancer patients [42]. ALBI was independently correlated with overall survival in multivariate analysis [42]. Other study also shown that the median progression free survival and median overall survival in low ALBI was longer than those in high ALBI in pancreatic cancer patients with liver metastasis [43].

In this study, we found that low ALBI was significantly related to longer PFS (*p* = 0.0002, HR: 3.039, 95% CI: 1.772–5.210) and OS (*p* = 0.0005, HR: 2.697, 95% CI: 1.539–4.720). The 1-, 3- and 5-year PFS and OS rates in low ALBI group were higher than those in high ALBI group. In the present study, underlying disease, liver metastases, CEA, AST, total protein, albumin, total bilirubin, direct bilirubin, creatinine and neutrophils were found to be significantly related to ALBI score. One study proved that liver status, CEA and CA199 were the important predictor in pancreatic cancer patients after pancreaticoduodenectomy [44]. Other study indicated that systemic inflammation response index, based on peripheral neutrophil, monocyte, and lymphocyte, was associated with pancreatic cancer patients survival, and could improve treatment outcomes by identifying candidates for active treatment [45]. According to univariate and multivariate analysis, the AST, alkaline phosphatase, prothrombin time and ALBI were the potential independent protective factor for PFS; and the AST, alkaline phosphatase, creatinine, prothrombin time and ALBI were the potential independent protective factor for OS. In the current study, multivariate analysis shown ALBI was an potential independent prognostic factor for liver metastasis of pancreatic cancer after pancreatoduodenectomy. Moreover, we constructed a nomogram by the potential prognostic factors by the multivariate analysis. The nomogram was used to predict the 1-, 3- and 5-year survival probabilities of PFS and OS. The calibration curve shown that the prediction line matched the reference line well for 3-year PFS and OS. Furthermore, the DCA shown that nomogram model was better than the only ALBI, and indicated the ability for clinical decision-making, especially in 1-year PFS, and 3-, 5-year OS.

The biological mechanism of ALBI as a potential prognostic factor of pancreatic cancer has not been clearly explained. We try to explain the relevance of each component of ALBI score. Albumin is composed in the liver, and can be used to reflect people’s nutritional condition [46]. The serum albumin concentration will be reduced when the nutritional condition is poor or liver function is impaired. The increase in bilirubin may be caused by biliary obstruction, and the impairment of liver can also cause the dysregulation of bilirubin. Moreover, the bilirubin can enter the brain interstitium freely and cause neurotoxicity.

The study has several limitations. Firstly, this study was a retrospective study, and a small number of patients were included in the study. And more of patients should be enrolled into study. Secondly, the small sample size is biased to guide the decisive conclusion of other potential prognostic factors. Finally, more homogeneous groups should be analyzed to validate this valuable conclusion in the future study. Therefore, multi-center and prospective studies should be enrolled to evaluate the prognostic value of ALBI score for pancreatic cancer patients after pancreatoduodenectomy with liver metastasis following radiofrequency ablation.

## Conclusion

ALBI is a potential independent factor for PFS and OS, and can predict the prognosis of pancreatic cancer patients after pancreatoduodenectomy with liver metastasis following radiofrequency ablation. Patients with low ALBI score have better prognosis and longer survival time. The nomogram model with ALBI may be a predictive stratification tool to facilitate clinical decisions.

## Data Availability

The raw data supporting the conclusion of this article will be made available by the authors, without undue reservation.
